# Geolocated dataset of Chinese overseas development finance

**DOI:** 10.1038/s41597-021-01021-7

**Published:** 2021-09-20

**Authors:** Rebecca Ray, Kevin P. Gallagher, William Kring, Joshua Pitts, B. Alexander Simmons

**Affiliations:** Boston University Global Development Policy Center, Boston, USA

**Keywords:** Conservation biology, Economics

## Abstract

China is now the world’s largest source of bilateral development finance and will likely continue to play a prominent role in sovereign lending through its multi-billion-dollar Belt and Road Initiative. This paper introduces major methodological enhancements in tracking this finance: the use of an original application programming interface (API) to gathers news in multiple languages; double-verification of every record to ensure every finance commitment has been formalized; and visual geo-location to trace the precise footprint of every project. The resulting dataset enables economic, environmental, and social analyses with high-precision spatial accuracy, as well as spatiotemporal monitoring by project stakeholders and enhanced planning by project managers. It covers the years 2008–2019 to enable analysis before and after the announcement of the Belt and Road Initiative. It includes 862 finance commitments, 669 of which have geographic location, to 94 countries across the world.

## Abstract (summary)

**Table:** 

Measurement(s)	finance record
Technology Type(s)	Application Program Interface
Factor Type(s)	geo-location • date • borrower category • sector
Sample Characteristic - Location	global • China

Machine-accessible metadata file describing the reported data: 10.6084/m9.figshare.14610678

## Background & Summary

Spatial analysis is a crucial tool in monitoring international development finance institutions’ (DFIs) footprint and impact^1–3^. This practice has become widespread as DFIs including the World Bank, Inter-American Development Bank, Asian Development Bank, and African Development Bank now publish project documents online, including location information. The World Bank publishes “geotags” with latitude, longitude and other project metadata, enabling analysis of their portfolio^4–6^.

China has now become a top source of development finance globally. Two DFIs account for the bulk of this finance: the China Development Bank (CDB) and the Export-Import Bank of China (ExImBank). In Latin America and the Caribbean, CDB and ExImBank together accounted for more committed sovereign finance than the World Bank from 2009 through 2018^7^. In Africa, for five of the years in that decade, CDB and ExImBank account for more finance committed than the World Bank^8^. Through its global infrastructure push—the Belt and Road Initiative (BRI)—China is likely to play an active role in international development finance for years to come. However, until now, researchers have been unable to extend to Chinese finance the same level of precision used in analysis of other DFIs, as CDB and ExImBank do not publish detailed records of their activities, and previous datasets have not been able to apply double-verification and precise geo-locations to global data.

Previous scholars’ pioneering work on Chinese development finance in Africa^9^ and Latin America^10^ created public databases^7,8^ and developed the double verification standard for technical validation. The 2017 AidData database^11,12^ expanded the frame of reference to include Chinese development finance, commercial finance, grants, and technical assistance. AidData’s was the first database to incorporate project locations, enabling global spatial analysis;^4^ this data was a crucial step for tracking China’s overseas development finance footprint, yet the lack of double verification methods and post-2014 data impedes our ability to fully grasp the magnitude of contemporary Chinese development finance.

Other scholars have examined additional facets of Chinese overseas development finance: infrastructure networks^13,14^, aggregate financial and debt implications^15–17^, investment and construction contracts^18,19^, and networks of construction and extraction projects^20^. These important aspects from prior efforts—including the double verification standard^7,8,21^, geolocation^11,12^, and aggregate mapping—provided the foundation of the present work.

This dataset introduces major improvements in three areas: project identification, verification, and geolocation. Our *project identification* process incorporates an original application programming interface (API) to gather news in multiple languages. *Double verification* ensures that every finance commitment has been formalized, rather than simply announced. Through additional *visual geolocation*, we introduce a newly stringent standard for spatial precision codes. In contrast to earlier datasets, the present dataset requires visual confirmation of each project’s footprint. We combine this information with project-specific attributes to enable analysis of economic and policy trends. Under this rigorous and innovate approach to verification, users can rely on the existence of each project, its attributes, and its precise geolocation.

This new dataset aims to empower stakeholders to monitor ongoing projects and to weigh potential risks and benefits of proposals. As the world faces rapid biodiversity losses and an impending climate crisis, this type of monitoring is more crucial than ever^22^. The United Nations and other global bodies have called for international DFIs to make radical shifts in the types of projects supported and the locations chosen for them. This dataset will enable tracking of progress toward those goals by including major DFIs that, until now, have not been fully traceable^23^, and facilitating research to evaluate potential environmental and social impacts of global Chinese development finance using high-precision spatial analyses^24^.

## Methods

Our aim has been to create a global, validated dataset of China’s overseas development finance from 2008 to 2019. We include all sovereign lending commitments by China’s two policy banks that are most active in overseas lending, CDB and ExImBank because as policy banks, they are differentiated from other sources of finance by their aim of supporting Chinese policy goals rather than commercial aims. The resulting trends can be interpreted as both economic and policy actions, akin to other policy-driven actors like multilateral development banks, national development banks, and export credit agencies^25^. As the interest rates associated with individual loans are far from universally public, our approach allows users to have a high degree of precision that the financing tracked here is extended in service of development policy aims.

To date, no official global aggregate or record of CDB and ExImBank overseas sovereign lending exist. Bank annual reports include lending for overseas projects, but include in those figures lending to Chinese companies for their work overseas and lending to private firms abroad, and in some cases include Chinese territories in overseas finance. As explained in more detail below, existing third-party aggregations largely fall into two categories: those that are not limited to DFIs and/or do not disaggregate by lender, and those that do not employ rigorous data validation to eliminate over-counting. For this reason, our methodology is bottom-up in nature, starting from individual loans and building to global aggregation.

**Fig. 1:**
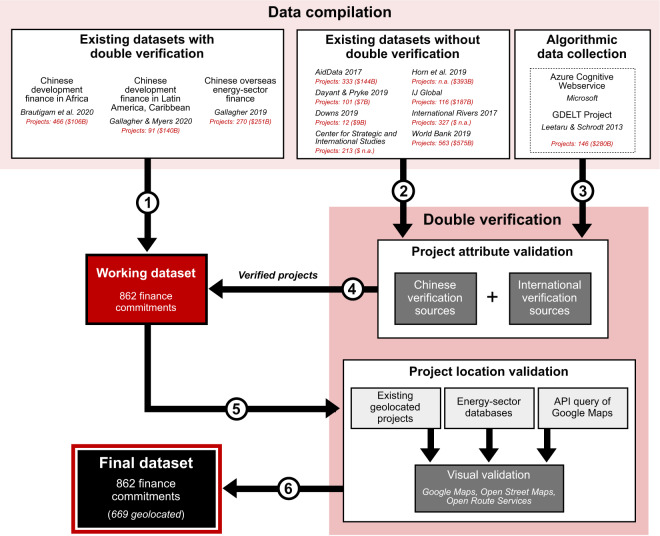
Process of compiling and validating records of China’s overseas development finance. Numbers indicate sequential steps, as described in the text. Note: Projects and amounts listed correspond to the observations in each source that would qualify for inclusion in the present dataset: sovereign finance commitments of $25 million USD or more, by CDB or ExImBank, between 2008 and 2019. The sum for Horn *et al*. (2019) reflects total debt from all Chinese lenders. The number of World Bank-reported projects reflects all named projects in the geo-located dataset. n.a. denotes data that is unavailable because it is not collected by the individual sources.

We applied a uniform validation standard of double verification (described in detail below) to every record incorporated here. This process had three steps: 1) we compiled the limited number of existing datasets of Chinese development finance that already meet this double-verification standard; 2) we then applied this standard to ‘clean’ other existing datasets, and 3) we created our own algorithm to unearth projects in countries and years that were not discovered in steps 1 and 2, and then validated these newly-discovered records through the double-verification method. These steps are illustrated together in Fig. 1.

### Step 1) Compiling existing doubly verified datasets

Three datasets already meet the double-verification standard and have been incorporated in their current state, though most do not have data through 2019: Brautigam *et al*. (2020)’s compendium of Chinese development finance commitments in Africa; Gallagher and Myers (2020)’s record for Latin America and the Caribbean; and Gallagher (2019)’s database of Chinese overseas energy-sector finance all meet this criteria^7,8,21^. Figure 1 shows the total number of records considered from each of these sources, as well as the final dataset size. While thousands of loans are considered from the various input datasets, and an additional 146 were discovered through our in-house news collection algorithm, the double-verification method narrows that universe to the 862 validated projects included in the final dataset.

### Step 2) Incorporating records from datasets without double-verification

We expand on these previous databases by applying the double-verification standard to Chinese development finance records from other existing databases. These datasets include:

- Bluhm *et al*., 2018^12^
- CSIS, n.d^13^.
- Dayant and Pryke, 2019^26^
- Downs, 2019^27^
- Horn, Reinhart, and Trebesch, 2019^15^
- IJGlobal, n.d^28^.
- International Rivers, 2017^29^
- World Bank, 2019^14^

The double-verification method is explained in greater detail in the technical validation section, below.

### Step 3) Identification of gaps and dataset completion through algorithmic data collection

Combining the coverage of existing datasets yields a patchwork of coverage, as Table 1 shows. Even if all of these observations could be validated, significant gaps would remain. Because of the significant gaps left among these datasets, and to ensure inclusion of projects that were not captured by existing efforts, we complemented these sources with our independent data gathering.

**Table 1:** Geographic, Sector, and Chronological Coverage of Existing Datasets of Chinese Overseas Development Finance.

	Region	Years	Sector
AidData, 2017	World	2000–2014	All
Brautigam *et al*., 2020	Africa	2000–2018	All
CSIS, no date	Asia	2006–2020	Infrastructure
Dayant and Pryke, 2019	Oceania	2007–2019	All
Downs, 2019	Pakistan	2006–2017	All
Gallagher, 2019	World	2000–2019	Energy
Gallagher and Myers, 2020	Latin Am., Carib.	2005–2019	All
Horn, Reinhart, and Trebesch, 2019	World	1949–2017	Aggregate lending
Hurley, Morris, and Portelance, 2018	68 borrowers	End-2016	Debt sustainability
IJ Global, no date	World	2008–2019	Infrastructure
International Rivers, 2017	World	2000–2017	Dams w/Chinese constr.
Kratz, Feng, and Wright, 2019	World	2011–2019	Debt renegotiation
MERICS, 2018	Asia	2013–2018	Rail, ports, pipelines
Scissors, 2020	World	2005–2020	FDI, construction
World Bank, 2019	Asia	2015–2018	Infrastructure

As of 2019, there is no singular source of truth for historical news access online. There are many public news aggregators, such as Google News and Apple News, which provide free or low-cost news searches. These aggregators also provide “real-time” feeds of news for users in their native language, with a preference for local results. For example, a search term of “baseball scores” performed in Boston will yield different results than the same search performed in San Diego; These would yield scores for Red Sox and the Padres, respectively. Many such news aggregators also provide historical searches, allowing users to select date ranges for a search term. Since these news aggregation services rely upon a combination of scraping web-based news sources and news licensing agreements, the quality and availability of these historical searchers are dependent on the publication quality and availability of source news^30–33^. In addition, there are private companies that provide similar access to historical news, often sourcing news through distribution agreements. These, too, vary widely in quality and breadth of coverage. Some of these include webhose.io, the GDELT project^34^, Lexis Nexis Uni, Factiva, among many others.

In political science, economics and other social sciences, it is now commonplace to utilize digital media and online news databases for data analysis^35–38^. Indeed, prior work around Chinese development finance mentioned above utilizes online news sources^39^. However, there remain a number of open questions around how to utilize online news databases as well as selecting for validity and reliability. For one, relying on only one source may introduce any number of data issues. In prior work, Blatchford (2020) explores the potential methodological weaknesses in utilizing a single online news source database for analysis^40^. Other issues may include discrepancies between news database sources^41,42^, the possible gatekeeping nature of news aggregators, as well as inconsistent or incomplete coverage^43^. We mitigate these issues by utilizing triangulation among multiple database sources, as well as first-level human validation, and the subsequent double verification. This blending of manual and algorithmic methods typically yields superior results^44,45^.

In order to algorithmically collect data, news aggregators and historical news services typically provide an Application Programming Interface (API) to facilitate programmatic access. This allows many thousands of individual searches (e.g. “China Development Bank loan” in “English” for “August-1-2015” yielding 50 results) to be performed by an algorithm. An algorithm collecting this data may take several hours to days to collect the entire corpus of search results for complex projects but will surpass a manual approach, which would take significantly longer and may introduce human errors^46^.

To select the news database sources we would use, we selected sources that provided an API and then tested them with the following methods. Each was tested using search terms such as ‘“China Development Bank” +loan’ to try to “organically” discover the news articles. This method is essential to verify that the approach will scale appropriately to other terms and still yield relevancy. To elaborate, we do not want to simply search for test terms such as ‘“China Development Bank” +“Thar Energy” +2018 +330 MW’ as these return desired results and imply 100% coverage. Rather, our goal is to identify terms that will maximize accuracy and coverage while also reducing the cost (most importantly, the required human-hours to verify and vet the resulting data from the algorithmic data collection). For example, using broad search terms, we encountered over 22,000 results from one year for one country. This would require over 360 hours of manual assessment at one minute per article. Our goal was to adjust the data collection parameters and post-process the collected data to reduce false positives as well as reduce duplicates so that the resulting effort by human-time will be minimized without a drop in accuracy or coverage. We utilized an established dataset of 2018 Energy Financing Projects to benchmark news databases against to measure coverage.

For this project, two data sources were selected and utilized in order to maximize data coverage. The first, Azure Cognitive Webservice^47^ is provided by Microsoft and has excellent coverage and is accessed by an API. One weakness of this API is the coverage, which is strongest around English articles. To supplement this, we utilize the GDELT project^34^ which has much greater coverage, especially for non-English languages, but yields significantly higher false positive matches. These false positives must be vetted manually, so a primary reliance on this data source would be untenable. A manual sampling of 500 shows that more than 85% of these are not relevant to the project and are indirect references. Contrast this to the Azure News service, which automatically sorts results by relevance; of the first 250 results, 95% are relevant to the project. These two databases combined provided over 95% coverage of known projects.

In this step, we utilized the following search terms:

- “China” or “Chinese” *and*

- “Development Bank,” “CDB,” “Export-Import,” “Export Import,” “ExIm,” “Ex-Im,” “Ex Im,” or “Eximbank” *and*
- The name of at least one borrowing country, in English or in other languages commonly spoken in each, in noun or adjectival form (for example “Iceland,” “Ísland,” “Icelandic,” or “Íslendingar” for Iceland; or “Hungary,” “Magyarország,” “Hungarian,” or “Magyar,” for Hungary).

Our scraping algorithm collected over one million records. Additional algorithms were created to further reduce the data by filtering to remove duplicates, poor news sources, and to remove articles which did not mention the key terms above in close proximity with one another. This process yielded 98,978 records. These were first manually scanned for relevancy and further reduced. Then, researchers manually checked and read each of the remaining records against existing records already included in the datasets listed above, resolving any conflicts in project attributes. Where conflicts arise between verification sources, we give government sources top priority, followed by academic sources, civil society sources, and private press sources.

### Steps 4–6) Technical validation

After we compiled these records, we subjected them to a multi-layered process of technical validation, described in more detail in the technical validation section, below. These validation stages apply harmonized definitions across the entire dataset, with particular focus on validation of finance commitment attributes (the data records described in the following section) and project location.

As many other authors have noted, this subject area is characterized with very low transparency^9,11,15^. Thus, while our method of double verification precludes the possibility of over-counting, it is still possible that some projects may be omitted due to insufficient public information. For this reason, all of the above steps are repeated annually, and in each update all years are included through the year prior to the update, in order to find projects that may not be reflected in public records for several years after financing is signed. In doing so, we join previous scholars including Brautigam *et al*. (2020) and Horn, Reinhart, and Trebesh (2019) in recognizing the importance of regular updates for maximum transparency in this intrinsically opaque field^8,15^.

## Data Records

The following information is gathered for inclusion in the final dataset.

### Project index

This unique code differentiates projects from each other and corresponds to observations mapped in the accompanying shapefile.

### Project name (English)

Projects are named in English, giving priority to include reference to local place names were possible.

### Country name and ISO

In the vast majority of observations, commitments are signed with individual countries, which are reflected here by name and by ISO 3166-1 alpha-3 codes. Thirteen additional finance commitments went to regional multilateral bodies, such as the Development Bank of Central Africa and the African Export-Import Bank.

**Fig. 2:**
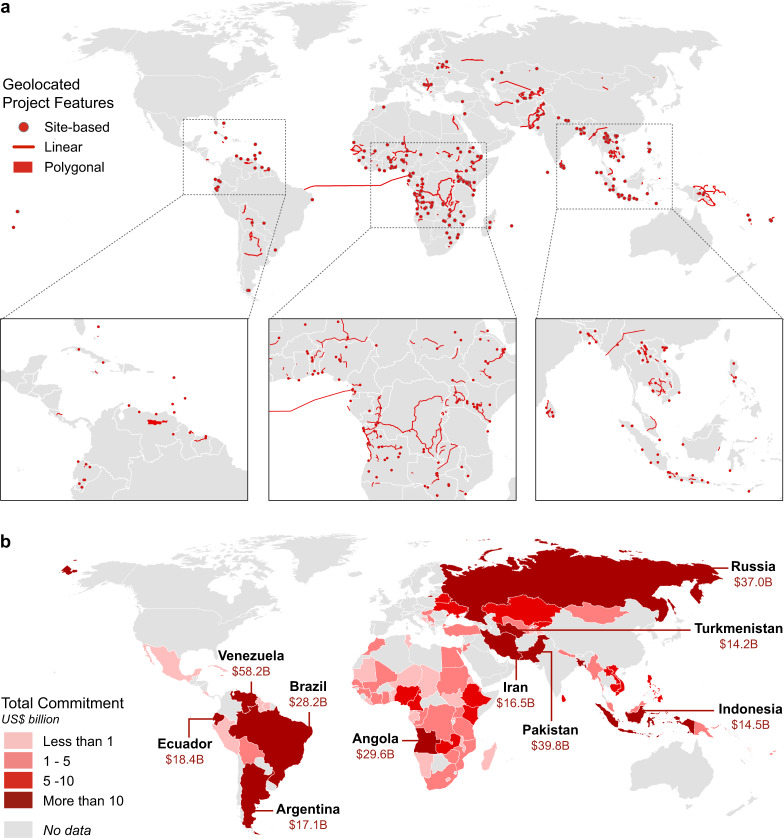
Locations of Chinese Development Finance Projects, 2008–2019. Figure 1a shows the locations of 669 projects with geographic footprints. Figure 1b shows national totals of all 862 financing commitments. The top ten recipient countries are indicated with individual labels.

As Fig. 2 shows, Chinese development finance is distributed widely across the world. Figure 2a shows the geographic footprints of the dataset, with special detail in three regions of particularly heavy representation: northern South America and the Caribbean; Sub-Saharan Africa, and Southeast Asia. Figure 2b shows national totals for finance commitments, and demonstrates that despite the broad coverage, a few countries comprise the bulk of the records. In fact, the top 10 recipients, labeled in Fig. 2b, comprise $277 billion in finance commitments, or 60 percent of the total.

### Borrower

All of the commitments here are to governments or entities wholly or partially owned by governments, including state-owned enterprises (SOEs), multilateral organizations, sub-national governments, and public-private partnerships. Where entities other than national governments are known, these are listed individually. Otherwise, all national government bodies (including ministries of finance, central governments, and other specifications) are listed as “national government,” for the sake of comparability among projects.

### Borrower category

Borrowers are shown in one of five categories, or combinations thereof:

- National government
- Public-private partnership
- Regional body
- State-owned entity
- Sub-national government

### Year signed

This column corresponds to the year that loan agreements were signed. It is important to note that this year does not necessarily correspond to the year when project construction was begun or completed. In many cases, financing is secured relatively late in the project cycle, long after letters of intent (LOIs) or memoranda of understanding (MOUs) are signed. In other cases, the reverse is true, and financing is secured well before a contractor is selected and final plans drawn up. Because the central actors in this data are the CDB and ExImBank, we reflect the year when they committed to financially support a project.

### Lender

The finance institution is shown as CDB or ExImBank. In five cases, co-financiers are shown, including the Bank of China, Citic, and the Industrial and Commercial Bank of China. In these cases, it was impossible to disaggregate the finance commitment among these lenders.

### Amount

In almost all cases, this amount shows the total committed by CDB and ExImBank, in millions of USD. However, in five cases of joint projects with other lenders, it was impossible to differentiate between the finance provided by these two and their co-financing partners. Those projects are noted with an asterisk in the next variable, Total Includes Co-Financing.

### Flag for Co-Financing

The five projects for which the listed finance commitments include co-financing from other banks (as described above) are indicated here.

### Sector

Projects are classified into one of ten major sectors, as follows.

- *Agriculture/food:* This category includes agriculture, fishing, and agricultural processing.
- *Extraction/pipelines:* This category includes mining, drilling, and pipeline transportation of extracted products. They are combined into one category because of the frequency of finance commitments that include both sub-sectors.
- *Government:* This category includes central bank operations (including foreign reserves), education, emergency response, environmental projects, government office construction, healthcare, national geological surveys, public housing, postal services, security, and national or regional development bank support.
- *Manufacturing:* This category includes refineries, smelters, and factories.
- *Power:* This category includes energy generation and distribution projects, which are further classified by the sub-sector or fuel (coal, gas/LNG, hydropower, nuclear, oil, solar, wind, distribution, other).
- *Telecommunications:* This category includes television, radio, wired and wireless communications networks, fiber optics and broadband data networks, satellite communications, and digitization and electronic dissemination of government services and records.
- *Transportation:* This category includes roads, airports, railways, and ports.
- *Water/wastewater:* This category includes irrigation, potable water, wastewater, and sewage projects.
- *Other construction:* This category includes industrial parks, business districts, shopping centers, athletic centers, conference centers, and general infrastructure commitments.
- *Multi-sector/discretionary:* This category includes finance commitments without specified purposes (for general budgetary support) or for projects that cross the sectors listed above.

As Table 2 shows, three sectors account for the bulk of China’s overseas development finance: transportation, extraction, and energy. Commitments in these three sectors came to $336 billion, or 72% of the total. A fourth important category of finance commitments includes those that cross multiple sectors or are discretionary (and able to be used as the borrower sees fit). Each of these sectors also includes trade finance for government purchases of related equipment, machinery, or vehicles.

**Table 2:** Sector Distribution of Finance Commitments by Year, Billions of USD.

	Transport	Extraction, Pipelines	Power	Multi-sector/Discretionary	Other	TOTAL
2008	1.2	0.4	3.4	0.6	1.9	7.5
2009	3.3	43.1	14.3	0.7	1.6	62.9
2010	12.7	0.0	5.1	20.3	3.1	41.2
2011	5.0	14.2	8.4	0.2	6.2	34.0
2012	8.0	2.2	9.6	4.2	10.9	34.9
2013	10.2	14.7	8.3	5.3	3.4	41.8
2014	22.6	5.0	13.4	4.0	3.7	48.6
2015	13.6	8.2	9.1	12.1	7.7	50.6
2016	21.7	20.7	7.7	4.5	20.3	75.0
2017	16.8	5.7	13.4	7.7	7.3	50.9
2018	4.6	0.0	4.3	0.0	4.1	13.1
2019	0.4	0.0	6.6	0.1	0.0	7.1

Note: “Other” includes Agriculture/Food, Government, Manufacturing, Telecommunications, and Other Construction. Sectors may not sum to the “Total” column value due to rounding.

### Validation links

As described in more detail below, the “double verification” method of data validation requires agreement between Chinese and international sources. Existing datasets using this standard of validation include Brautigam *et al*. (2020), Gallagher (2019), and Gallagher and Myers (2020)^7,8,21^. Records originating in these datasets are presented without further validation information. For all other records, Internet links for double verification are displayed for replication of this validation process. This inclusion allows users to assess the reliability of each record.

### Location

For the 664 projects with geographic location information, these are provided in the accompanying shapefile.

### Precision level

Following AidData^11,12^, we indicate the level of precision of our location data. As described above, our use of these codes differs from previous datasets, as follows:

1. Exact project footprint
2. Within 25 km, based on sources labeling a project as “near” a mapped place. This category also includes projects known at the second-order administrative division where that division has a radius of less than 25 km.
3. Second-order administrative division, such as municipality or county
4. First-order administrative division, such as state or province
5. Spanning multiple first-order administrative divisions
6. Country
7. Unknown

As Table 3 shows, the overwhelming majority of projects are located at the exact or “near” level: over 80% of commitments accounting for roughly 90% of committed finance.

**Table 3:** Precision Levels of Geolocated Finance Commitments.

Precision level:	Number of Commitments		Commitment Total (USDb)	
	Absolute	Percent	Absolute	Percent
Exact	460	68.8%	219.5	79.8%
Within 25 km	136	20.3%	31.8	11.6%
2^nd^ order A.D.*	23	3.4%	6.8	2.5%
1^st^ order A.D.*	28	4.2%	13.7	5.0%
Multiple 1^st^ order A.D.s*	4	0.6%	0.4	0.2%
Country	18	2.7%	2.7	1.0%
Unknown	0	0.0%	0.0	0.0%
**Total**	**669**	**100.0%**	**270.0**	**100.0%**

Note: A.D.: Administrative divisions within countries. First-order administrative divisions are often called states, provinces, or departments. Second-order administrative divisions are often called municipalities or counties.

The resulting records are available in two datasets – project attributes and project locations – at the Open Science Framework repository (10.17605/OSF.IO/GFWHJ). The former file, project attributes, includes all variables except for each project’s location, and is freely available in CSV and XLSX formats. The latter dataset, project locations, is freely available upon completion of the data use agreement, which is also available in the same repository. The project locations dataset includes separate files for point-based, linear, and polygonal projects, in Esri shapefile (SHP) formats^48^.

In addition, two sets of code are available at the same repository. News aggregation code is available in Python and geolocation code (querying Google Maps and Open Street Maps APIs) is available in R, upon completion of the same data use agreement mentioned above^48^.

## Technical Validation

This dataset relies on two types of technical validation: ensuring the accuracy of (1) project attributes and, where applicable, (2) their geographic locations.

### Project attribute validation: the double-verification method

Existing sources for Chinese overseas development finance rely on a variety of verification standards. The present dataset extends the most stringent approach of the existing “double verification” methods pioneered by the China Africa Research Initiative at the Johns Hopkins University School of Advanced International Studies (SAIS-CARI) to create a harmonized, global standard.

The double verification method is based on academic literature showing a tendency to overstate, rather than understate, finance commitments. For example, Ebeke and Ölçer^49^ show that major infrastructure projects are often timed for announcements to coincide with political campaigns. Regional case studies^9,50^ show patterns of planners avoiding the publication of projects’ environmental and social risks, but simultaneously maximizing the visibility of the projects and their financial commitments, often before they are finalized. For this reason, earlier datasets have struggled to correctly identify and exclude projects that have been publicized but never materialized, resulting in sometimes significant over-estimations^51^.

The possibility remains of under-counting. As Horn, Reinhart, and Trebesch (2019)^15^ point out, in reference to “hidden” Chinese finance, many overseas Chinese loans are never fully disclosed. For this reason, we cast the widest possible net for financing commitments and then narrowing those findings by applying the standard of double-verification. It is for this reason also that we perform annual updates, and in each update include previous years’ data, in order to include any additional projects that may not have been disclosed until a much later date.

Our aim is to provide the most evidence-based supported data in order to have a more empirical based understanding of Chinese overseas development finance. Erring on the side of caution then, double verification is admittedly a more conservative set of estimates but grants all scholars and stakeholders the confidence that every record in the dataset does indeed exist.

Without public reporting by CDB and ExImBank of their lending operations, we are limited to reporting by government (and government-affiliated) sources, academic, civil society, and press reports. The system of double verification ensures accuracy in this context, requiring agreement on the core characteristics of each loan agreement between at least one Chinese source and at least one international source.

For China-side verification, we rely on official and quasi-official sources associated with the Chinese government or Chinese Communist Party. We include the following sources:

1. Chinese government and DFI websites (including CDB.com.cn, ExImBank.gov.cn, and any other source with a domain ending in .gov.cn)
2. Websites of Chinese embassies abroad
3. Chinese government or CCP-affiliated press sites:
  - *China Daily*, http://www.chinadaily.com.cn
  - China Global Television Network, https://www.cgtn.com
  - *China News*, http://www.chinanews.com
  - *China Plus*, http://chinaplus.cri.cn
  - *Guangming Daily*, http://www.gmw.cn
  - *People*, http://www.people.cn
  - *Xinhua*, http://www.xinhuanet.com

For international verification, we rely similarly on government reports, supplemented with academic, civil society, and private press reports. As mentioned above, when differences emerge among sources, we resolve these conflicts by giving government sources top priority, followed by academic sources, civil society sources, and private press sources. Government press sources, such as the Chinese sources listed above, are given the weight of government sources. This method coincides with that of other datasets with double verification^7,8,21^.

Because of the stringency of the double-verification standard used here, we exclude the smallest finance agreements (those below $25 million USD). Excluding these low-level loans necessarily involves a small degree of under-counting. For example, Brautigam *et al*. (2020)^8^ show that loans of less than $25 million each comprise just $389 million in total commitments, out of a total of $148 billion in financing commitments by CDB and ExImBank between 2008 and 2018 in Africa: approximately 0.2% of the total. However, including these loans would introduce significant geographic bias toward countries with particularly transparent governments and open media environments. As the purpose of the present effort is to enable more reliable geospatial analysis, the inclusion of this additional activity was not deemed worthy of the cost to the reliability of analysis using it.

It is worth comparing these results to those of other datasets for context. Among other independent datasets of Chinese lending, only AidData^11,12^ and Horn, Reinhart, and Trebesch^15^ have global coverage, and of those two, only AidData differentiates by lender, allowing a strict comparison. As Fig. 1 shows, AidData includes $463 billion in policy bank loans between 2008 and 2014 that would meet the standard for inclusion in the present dataset if they could be validated. However, in that same time period, our methodology found that only $271 billion of loans could pass the validation standards introduced here.

This process of double-verification results in a dataset that excludes some countries that appear in other datasets. For example, in the case of four countries, this process resulted in the present dataset having no loans listed, even though CDB and/or ExImBank loans appear in AidData, the largest global dataset, with loans that would qualify for inclusion here if they could be validated. Those four are: Central African Republic (for which we were unable to find doubly verified validation for the Boali No. 3 hydropower plant project), Dominica (for which we were unable to double verify the source of the loan for rehabilitation of State College), Turkey (whose Turk Telecom was privatized before the loan listed in AidData), and Yemen (for which we were unable to find Chinese validation for the Bajal cement factory project). In addition to these four countries, three others are included in AidData but with no loans of $25 million or more: Burundi, Colombia, and Sierra Leone.

As with other researchers in this space^7,8,21^ we understand that individual projects within such funds can be hidden from public view until the line of credit or framework agreement is renewed or laid down unused. Thus, we include such financing agreements when they are initially drawn up, but then withdraw them from subsequent updates if it comes to light that they were unused. If they are renewed, as lines of credit frequently are, such renewals do not represent new financing but simply a relaxation of the time period for use of the original commitment. For this reason, renewals are not considered separately.

Finally, not all projects in this dataset have been completed as of this writing. We have removed all projects that have been publicly cancelled, but ongoing projects with active financing commitments remain, even if construction has not yet begun or has been suspended. For this reason, we refer to each observation as a commitment or agreement, rather than a loan. Funds may or may not have been disbursed as of this writing, but commitments have been made and remain valid. In all, this double-verification process resulted in a final dataset of 857 finance commitments in 93 countries from 2008 through 2019.

### Location validation

Of the 857 finance commitments in the final dataset, 664 have a geographic footprint of some type. These projects – encompassing agriculture, extraction, manufacturing, utilities, infrastructure, and other installations – were located according to the following procedure.

Several of the existing datasets listed above include the location of financed projects: AidData, CSIS, Dayant and Pryke, and the World Bank^11,13,14,26^. Among these datasets, CSIS’ Reconnecting Asia merits special mention, as it displays project locations through embedded Google Maps. For projects originating in this dataset, we queried CSIS for the coordinates in these maps (using code available in R as CSIS_to_coord_str.R on the project repository). For these observations, we used these reported locations as initial estimates, to be visually validated thereafter. For energy projects not listed in these project datasets, we used the following sources for initial estimates of project locations:

- Power plants: Global Power Plant Database^52^.
- Coal-fired power plants: Global Energy Monitor^53^
- Fossil fuel pipelines and related infrastructure: Global Fossil Infrastructure Tracker^54^

For other observations, we developed an API to query Google Maps for the locations of each (available in R as GoogleMaps_OSM_API_query.R on the OSF project repository).

**Fig. 3:**
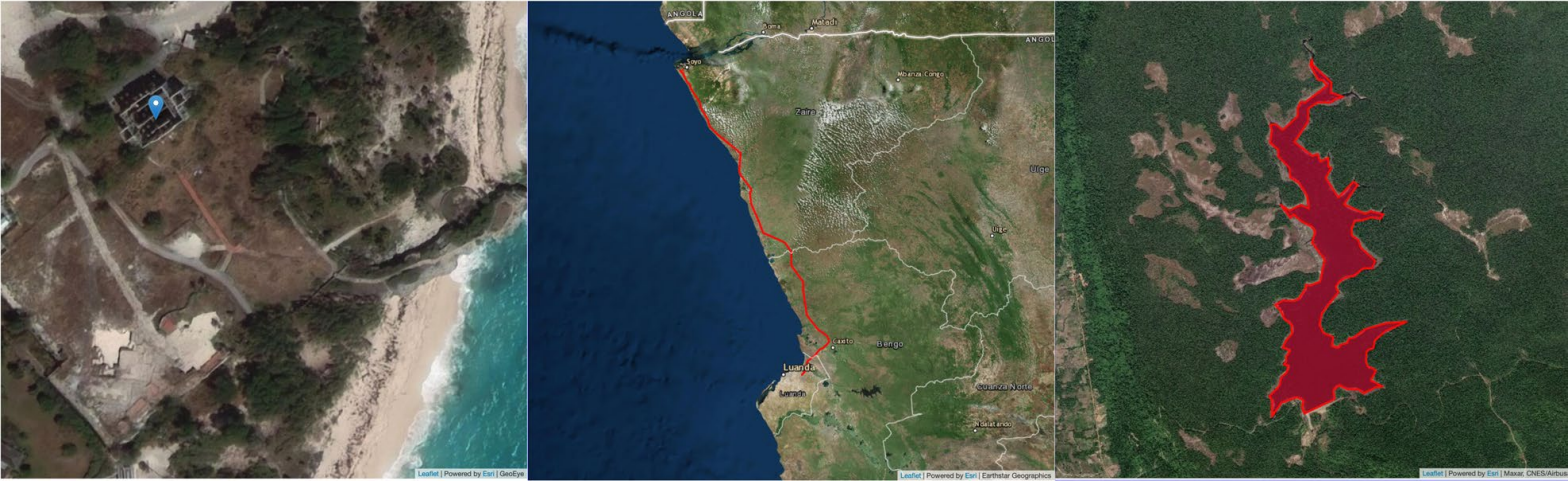
Examples of point, line, and polygon footprints. Left to right: Rehabilitation of Sam Lord’s Castle, Barbados; Soyo-Kapary Electrical Transmission and Transformation Project, Angola; Kirirom III hydropower plant (reservoir), Cambodia.

For all observations – those included in previous geolocated datasets, those located through querying Google Maps and Open Street Maps, and those with no query response – we validated the locations visually through the use of Google Maps, Open Street Maps, and Open Route Services, as shown in Fig. 3 below.

This process represents a significant elevation of requirement needing to be met for projects to be reported as having a precise location, in comparison to previous geocoded datasets. For example, AidData allows projects to be reported at the most precise location category based on the precise boundaries of an area of uncertainty around a project—including populated places or the political seats of geographic areas—rather than the precise point or boundaries of the true project site(s). The resulting high-precision category includes 579 sovereign finance commitments by CDB and ExImBank identified by AidData during our period of study, of which only 105 geotags are associated with specific sites of projects. The remaining projects’ location are defined by the administrative division or the political seats thereof. This is in contrast to the more stringent precision classification scheme in our dataset. Projects marked with a precision code of “1” in the present dataset have all been visually located as site-specific project footprints. The introduction of this new level of precision allows for linear and polygonal projects to be represented with their complete footprints, rather than representative points, which enables a more thorough analysis of environmental risks and impacts, including for example, the impacts of the entire length of a highway or the entire area of a mine. Analysts using this dataset will be able to avoid the under-estimation of environmental impacts necessarily introduced by relying on representative points. Our first such analysis uses these precise footprints to compare location-based social and ecological risks of Chinese overseas development finance to World Bank projects, based on their proximity to the boundaries of national protected areas, possible critical habitats, and indigenous territories^48^. The dataset also supports holistic environmental analysis of interconnected networks of projects, based on their collective footprints. Yang *et al* (2021) use these collective footprints to examine the environmental and social sensitivity of Chinese overseas development finance locations, and find that the total footprint is significantly concentrated in more sensitive territory than World Bank projects during the same time period^55^.

To accurately reflect the variety of types of footprints across various types of finance projects, we classified each geolocated observation as a point (or collection of points), line (or collection of discontinuous lines), or polygon (or collection of discontinuous polygons). Points are used for individual buildings or installations. Lines are used for linear infrastructure including roads, rails, power distribution, wired communications networks, and pipelines. Polygons show projects with footprints that are larger than single buildings or installations, with well-defined boundaries, including dam reservoirs, oil and gas fields, and clusters of buildings such as housing or stadium complexes. The distribution of projects among footprint types is listed in Table 4.

**Table 4:** Footprint types.

Footprint types	Number of Commitments		Commitment Total (USDb)	
	Absolute	Percent	Absolute	Percent
No footprint	193	22.4%	192.4	41.2%
Point(s)	283	32.8%	127.2	27.2%
Line(s)	256	29.7%	117.0	25.0%
Polygon(s)	130	15.1%	30.8	6.6%
Total	862	100.0%	467.3	100.0%

A few examples merit further explanation regarding their classification of footprint type. First, wind farms are comprised of turbines along access roads; to accurately show the total geographic footprints, we show them as linear infrastructure comprised of their access roads. In addition, projects with lower levels of geographic precision (at the national level or first/second-level administrative division level) are shown as polygons that encompass these areas, showing the municipal, provincial, or national boundaries^48^.

## Data Availability

Two sets of code are available in conjunction with the resulting data, at 10.17605/OSF.IO/GFWHJ. News aggregation code is available in Python. Geolocation code (querying Google Maps and Open Street Maps APIs) is freely available, in R, upon completion of the data use agreement, which is also available in the same repository^48^.
